# The Global Regulator PhoU Positively Controls Growth and Butenyl-Spinosyn Biosynthesis in *Saccharopolyspora pogona*

**DOI:** 10.3389/fmicb.2022.904627

**Published:** 2022-06-09

**Authors:** Jianli Tang, Jianming Chen, Yang Liu, Jinjuan Hu, Ziyuan Xia, Xiaomin Li, Haocheng He, Jie Rang, Yunjun Sun, Ziquan Yu, Jun Cui, Liqiu Xia

**Affiliations:** Hunan Provincial Key Laboratory for Microbial Molecular Biology, State Key Laboratory of Developmental Biology of Freshwater Fish, College of Life Science, Hunan Normal University, Changsha, China

**Keywords:** PhoU, butenyl-spinosyn, *Saccharopolyspora pogona*, global regulator, phosphate

## Abstract

Butenyl-spinosyn, a highly effective biological insecticide, is produced by *Saccharopolyspora pogona*. However, its application has been severely hampered by its low yield. Recent studies have shown that PhoU plays a pivotal role in regulating cell growth, secondary metabolite biosynthesis and intracellular phosphate levels. Nevertheless, the function of PhoU remains ambiguous in *S. pogona*. In this study, we investigated the effects of PhoU on the growth and the butenyl-spinosyn biosynthesis of *S. pogona* by constructing the mutants. Overexpression of *phoU* increased the production of butenyl-spinosyn to 2.2-fold that of the wild-type strain. However, the *phoU* deletion resulted in a severe imbalance of intracellular phosphate levels, and suppression of the growth and butenyl-spinosyn biosynthesis. Quantitative Real-time PCR (qRT-PCR) analysis, distinctive protein detection and mass spectrometry revealed that PhoU widely regulated primary metabolism, energy metabolism and DNA repair, which implied that PhoU influences the growth and butenyl-spinosyn biosynthesis of *S. pogona* as a global regulator.

## Introduction

*Streptomyces* are gram-positive bacteria that produce a variety of secondary metabolites used in the pharmaceutical and agricultural industries, including various anticancer agents, antibacterial agents, antibiotics and biological insecticides (Khan et al., 2011; Lucas et al., 2013). Butenyl-spinosyn is an environmentally friendly biological insecticide with great development prospects produced by *Saccharopolyspora pogona*, which has activity against the worldwide quarantine pests *Laspeyresia pomonella* and *Helicoverpa assulta* (Darriet et al., 2005; Dos Santos Dias et al., 2017). However, the yield of butenyl-spinosyn is too low to be industrially produced on a large-scale. Therefore, it is urgent to overcome the technological barrier by constructing mutants with high butenyl-spinosyn production.

Phosphorus plays an important role in the process of life (Solans et al., 2019). In addition, the researchers have found that the biosynthesis of most natural products produced by actinomycetes is modulated by the concentration of phosphate in the medium (Romero-Rodriguez et al., 2018). Studies have shown that a high concentration of phosphate (above 10 mM) leads to a decrease in the production of antibiotics, as occurs in *S. griseus*, *S. peucetius*, and *S. clavuligerus* (Santos-Beneit, 2015). Conversely, low phosphate levels has been shown to promote the transition phase to aerial mycelium generation (Tenconi et al., 2012)and the biosynthesis of secondary metabolites (Rodriguez-Garcia et al., 2007). Recently, Rang et al. (2020) found that the growth of *S. pogona* and the biosynthesis of butenyl-spinosyn were inhibited to a certain extent with the an increase in phosphate levels in the culture medium. There results suggested that phosphorus, as an essential element for organisms, plays a key role in regulating the growth and the biosynthesis of secondary metabolites.

Previous studies have indicated that there are two-component systems involved in phosphate regulation, one of which is the PhoR-PhoP system, which is extremely conserved and widely present in actinomycetes (Santos-Beneit, 2015; Aggarwal et al., 2017; Brokaw et al., 2017). Additionally, a pivotal protein named PhoU is involved in phosphorus sensing (de Almeida et al., 2015; Brokaw et al., 2017; Shang et al., 2020). The function of the PhoU protein is mainly exerted by regulating the uptake of phosphate mediated by the Pst system and the phosphate restriction response mediated by the PhoR-PhoP two-component system (diCenzo et al., 2017). PhoU is mainly involved in regulating the uptake of inorganic phosphate in the environment, and plays an important role in the balance of phosphorus metabolism as a regulatory protein of the phosphate transport system (Martin-Martin et al., 2018). For example, mutation of PhoU was found to lead to a metabolically hyperactive status of the cell, as shown by increased expression of energy production, chemotaxis and flagella genes in *Escherichia coli* (Li and Zhang, 2007). Deletion of the *phoU* homologous genes *phoU1* and *phoU2* in *Staphylococcus aureus* led to the upregulation of inorganic transport genes and an increase in intracellular polyphosphate (polyP) levels (Shang et al., 2020). Moreover, PhoU not only affects cell growth and metabolism, but also participates in the regulation of the biosynthesis of secondary metabolites of Streptomyces. Martin et al. isolated a strain of Streptomyces coelicolor with a *phoU* gene deficiency, and through phenotypic analysis, they found that the mutant strain grew slower than the wild-type strain, had weak spore germination capacity and lacked pigment. Furthermore, the deletion of the *phoU* gene also was shown to lead to a decrease in the production actinorhodin (ACT) and undecylprodigiosin (RED) (Martin-Martin et al., 2018). Kim et al. (2018) also found that the *phoU* and *afsS* genes in *S. coelicolor* have a regulatory effect on the secondary metabolites ACT and RED and that this regulation is interrelated. Although PhoU has been studied in some pathogenic bacteria and type *Streptomyces*, few studies have focused on its effects and regulatory mechanism in *S. pogona*.

In this study, we constructed *phoU* overexpression and knockout strains to explore its effect on the growth and butenyl-spinosyn biosynthesis in *S. pogona*. The results showed that the deletion of *phoU* significantly changed the phenotypic characteristics of *S. pogona*, such as stunted growth and a decrease in butenyl-spinosyn yield. Overexpression of *phoU* could significantly increase the yield of butenyl-spinosyn and the insecticidal activity of the fermentation supernatant. To explore the possible reasons for these phenomena, the intracellular polyP and precursor levels, related gene transcription levels and distinctive proteins of the wild-type and mutant strains were determined. In general, the evidence above indicated that PhoU regulated the growth and development of *S. pogona* and the biosynthesis of butenyl-spinosyn by controlling intracellular phosphate levels and various primary metabolisms.

## Materials and Methods

### Bacterial Strains, Plasmids, Media, and Growth Conditions

The bacterial strains, plasmids, and primers used in this study are listed in Supplementary Tables 1, 2. *S. pogona* NRRL 30141 was cultivated in CSM medium (per liter: 10 g glucose; 45 g trypticase soy broth; 9 g yeast extract; 2.2 g MgSO_4_) with 280 r/min oscillation at 30°C for 48 h. Then, 2.5 mL of bacterial suspension was added to 50 mL of fermentation medium (per liter: 1 g KNO_3_; 0.01 g FeSO_4_; 0.5 g K_2_HPO_4_; 0.5 g MgSO_4_; 20 g glucose; 4 g yeast extract; 4 g tryptone; pH 7.2) and incubated at 30°C for 10 day. The recombinant stains were cultured under the same conditions with the addition of antibiotics (apramycin, 50 mg/L). Conjugative transfer between *E. coli* and *S. pogona* was performed using R6 medium (200 g/L sucrose; 10 g/L dextrin; 26 g/L BHI; 1 g/L casamino acid; 0.1 g/L K_2_SO_4_; 0.05 g/L FeSO_4_.7H_2_O; 0.05 g/L MgSO_4_.7H_2_O; 0.001 g/L MnCl_2_.4H_2_O; 0.001 g/L ZnSO_4_.7H_2_O; 0.01 mol/L MOPS; 0.048 mol/L CaCl_2_; 0.065 mol/L L-glutamic acid; 2% agar powder), and the conjugants were then incubated at 30°C for 10 day. *E. coli* was cultivated in LB medium, supplemented with antibiotics as required (apramycin, 50 mg/L), with 220 r/min oscillation for 12 h at 37°C.

### Construction of Recombinant Strains

To produce pOJ260-*P*_*kasO*_-*phoU*, the primer pair *phoU*-F/*phoU*-R was designed (Sangon, Shanghai, China) to amplify the *phoU* gene from *S. pogona* genomic DNA, and the primer pair *P*_*kasO*_-F/*P*_*kasO*_-R was designed to amplify the *P*_*kasO*_ gene from pUC57-Amp-*P*_*kasO*_. The amplified fragments were fused by overlap extension PCR with the primer pair *P*_*kasO*_-F/*phoU*-R. The fusion fragment was cloned into the corresponding restriction sites of the plasmid pOJ-260 after enzyme digestion (*Hin*dIII and *Eco*RI), and the recombinant plasmid pOJ260-*P*_*kasO*_-*phoU* was obtained (Supplementary Figure 1A).

The sgRNA was amplified by the primer pair *phoU*-sgRNA-F/*phoU*-sgRNA-R from pKCcas9dO, and the primer pair *phoU*-UHA-F/*phoU*-UHA-R and *phoU*-DHA-F/*phoU*-DHA-R were designed to amplify the upstream and downstream fragments of the *phoU* gene from the genomic of *S. pogona*. The amplified fragments were fused by overlap extension PCR with the primer pair *phoU*-sgRNA-F/*phoU*-DHA-R. Then, the fusion fragment digested by restriction enzymes (*Hin*dIII and *Spe*I) was cloned into the plasmid pKCcas9dO digested with the same enzymes (Supplementary Figure 1B).

The recombinant plasmid was introduced into the wild-type strain of *S. pogona* by conjugation, yielding the recombinant strains *S. pogona*-*phoU* and *S. pogona*-Δ*phoU* (Supplementary Figure 2).

### Cultivation Profile Analysis of the Wild-Type and Recombinant Strains

To detect the influence of the *phoU* gene on the growth and phenotype of *S. pogona*, yield assays, growth curve determination and morphological observations of the wild-type and recombinant strains were performed (Tan et al., 2017). During fermentation, 600 μL of fermentation supernatant was extracted by an equal volume of ethyl acetate in a water bath at 65°C every day, and 500 μL of the supernatant was lyophilized and dissolved in 50 μL methanol. Then, the dissolved sample was detected by HPLC (Tang et al., 2021b).

To monitor the insecticidal activity of butenyl-spinosyn against *H. armigera*, 2 mL fermentation supernatants of the wild-type and recombinant strains were mixed with 20 mL fodder (per liter: 70 g bean flour; 40 g yeast extract; 36% acetic acid; 5 g vitamin C; 15 g agar and 10 g penicillin) and evenly distributed in 24-well plates with one *H. armigera* per well. 3 parallel plates were set up in each experimental group, each parallel plate contained 24 *H. armigera*, and an equal volume of water was added to replace the fermentation supernatants in the control group. The death of *H. armigera* was recorded every day for 6 day (Tang et al., 2021a).

### Protein Extraction and Sodium Dodecyl Sulfate PolyAcrylamide Gel Electrophoresis (SDS-PAGE) Analysis

To monitor the difference in protein expression between the wild-type and recombinant strains, cells of different fermentation periods (2, 4, 6, and 8 day) were collected for whole protein extraction (Yang et al., 2014). The concentration of protein samples was detected with a Bradford protein concentration detection kit (Sangon, Shanghai, China), and then, 20 μg of each sample was analyzed by SDS-PAGE.

### Heterologous Expression and Western Blot Analysis of PhoU

To verify the expression of PhoU in recombinant and wild-type strains, the heterologous expression vector pCold-TF-*phoU* was constructed. The primer pair H-*phoU*-F/H-*phoU*-R was designed to amplify the *phoU* gene from the genome of *S. pogona*, and the fragment digested with *Hin*dIII and *Xba*I was cloned into the plasmid pCold-TF, yielding the recombinant vector pCold-TF-*phoU*, which was transferred to *E. coli BL21* (*DE3*). The recombinant strain was fermented in LB medium with 50 mg/mL ampicillin and induced to express PhoU protein by IPTG (Supplementary Figure 3A). The single PhoU protein was purified by Ni-NTA column after elution with different concentrations of imidazole buffer (Supplementary Figure 3B). The purified protein was injected into mice as an antigen, and then the expression of PhoU in recombinant and wild-type strains was detected *via* Western blot (Li et al., 2019).

### Liquid Chromatography-Tandem Mass Spectrometry Analysis

The distinguishing protein bands between the recombinant and wild-type strains and the heterologously expressed protein PhoU were excised from the SDS-PAGE gel for in-gel digestion (Supplementary Figure 6; Li et al., 2018). Subsequently, 30 μL of each digested sample were taken out separately and analyzed by liquid chromatography-tandem mass spectrometry (LC-MS/MS) with an LTQ XL mass spectrometer (Thermo Fisher, San Jose, CA, United States) (Yang et al., 2015).

### Detection of Intracellular Polyphosphate and Malonyl-CoA

Intracellular polyP levels were detected by using 4′6-diamidino-2-phenylindole (DAPI; Sigma) as previously described (Kulakova et al., 2011). *S. pogona* was cultured in CSM medium at 30°C for 48 h. Then, 2.5 mL of a bacterial suspension was added to 50 mL of fermentation medium and incubated at 30°C with 280 r/min. The fermentation broth at different time points (2, 3, 4, 5, 6 day) was removed and centrifuged (10,000 × *g*, 15 min) respectively, and the supernatant was discarded. The bacterial pellet was suspended in water to maintain a consistent OD_600_ value (about 2) for each sample. 1.5 mL of bacterial suspension was taken out, centrifuged at 10,000 × *g* at 4°C for 15 min, and the supernatant was discarded. The bacterial pellet was resuspended with 1 mL HEPES (50 mmol/L, pH = 7.5) and stored at −80°C for polyP quantification. The 300 μL processed samples were mixed with 600 μL HEPES buffer, then 100 μL DAPI solution (100 μM) was added to the buffer, and 100 μL H_2_O was substituted for DAPI solution in the control group. The binding of DAPI to polyP causes a shift in absorbance from 414 nm to 550 nm. The fluorescence signal was monitored by using a Microplate Reader (Thermo Scientific, United States) with excitation at 415 nm and emission at 550 nm, after 5 min of incubation at room temperature in the dark. Fluorescence values were normalized to background fluorescence of cells and HEPES buffer used as a control. Three biological replicates were performed for each sample to calculate the standard deviation.

The fermentation broth at different time points (2, 4, 6 day) was removed and centrifuged (10,000 × *g*, 15 min) respectively, and the supernatant was discarded. The cell pellet was diluted with PBS (pH 7.2-7.4) to bring the cell concentration to around 1 × 10^6^ CFU/mL. These samples were freeze-thawed repeatedly to disrupt cell walls and release intracellular components, then centrifuged at 3,000 × *g* for 20 min and the supernatant collected. The content of intracellular malonyl-CoA of different strains was determined using the microorganism malonyl-CoA ELISA kit (Shanghai Fangke Industrial Co., Ltd.) according to the instructions. Three biological replicates were performed for each sample to calculate the standard deviation.

### RNA Extraction and Quantitative Real-Time PCR (qRT-PCR) Analysis

One milliliter of the bacterial suspension after culturing for 6 day was taken out and centrifuged at 10,000 × *g* for 5 min, then the supernatant was removed and the bacterial pellet was washed three times with water, and stored at −80°C for extraction of total RNA. Total RNA from recombinant and wild-type strains was extracted by using TRIzol Reagent (Sangon, Shanghai, China) according to the instructions. The concentration of RNA was determined by monitoring the ratio of OD260 nm to OD280 nm on a NanoDrop 2000 spectrophotometer (Thermo Fisher Scientific, Waltham, MA, United States). DNase treatment and cDNA synthesis were carried out by using a Maxima H Minus First Strand cDNA Synthesis Kit with dsDNase (Thermo Fisher Scientific, Waltham, MA, United States). The above experimental methods were performed according to the relevant instructions. Real-time quantitative PCR amplification of RNA was performed by using SYBR^®^ Premix Ex Taq™ GC (Takara, Kyoto, Japan), and then, PCR was performed under the following conditions: 2 min at 50°C, 10 min at 95°C, and 40 cycles of 15 s at 95°C and 1 min at 60°C. The transcription level of related genes was monitored on a 7500 Real-Time PCR system instruments (Applied Biosystems, United States). 16S rRNA was employed as an internal control to quantify the relative expression of target genes. Three biological replicates for each sample were performed.

### Statistical Analysis

All the experimental results were statistically analyzed by using SPSS statistics version 17.0 software and stated as means ± standard deviation (SD). Student’s *t*-test, “*” *P* < 0.05, “^**^” *P* < 0.01, “^***^” *P* < 0.005, and “NS,” no significance.

## Results

### Verification of the PhoU Expression Level

**FIGURE 1:**
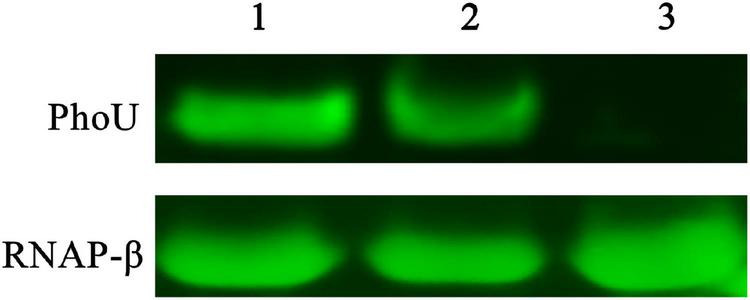
Western blot verified differences in PhoU expression levels between wild-type and recombinant strains. 1, 2, and 3 represent samples of *Saccharopolyspora pogona*-*phoU*, *S. pogona* and *S. pogona*-Δ*phoU*, respectively. A 20 μg whole protein sample of each strain was used separately for comparative analysis. RNAP-β was used as an internal reference protein.

To verify the expression of PhoU in recombinant and wild-type strains, Western blot was performed. PhoU was successfully harvested from *E. coli* BL21 (DE3) and purified *in vitro* (Supplementary Figure 3), followed by identification by 1D-LC-MS/MS. Subsequently, antibodies were produced by immunizing BALB/c mice. The Western blot results demonstrated that the expression level of PhoU in *S. pogona*-*phoU* was upregulated compared with that in *S. pogona*, while the expression of PhoU could not be detected in *S. pogona*-Δ*phoU* (Figure 1).

### PhoU Affects the Biosynthesis of Butenyl-Spinosyn

**FIGURE 2:**
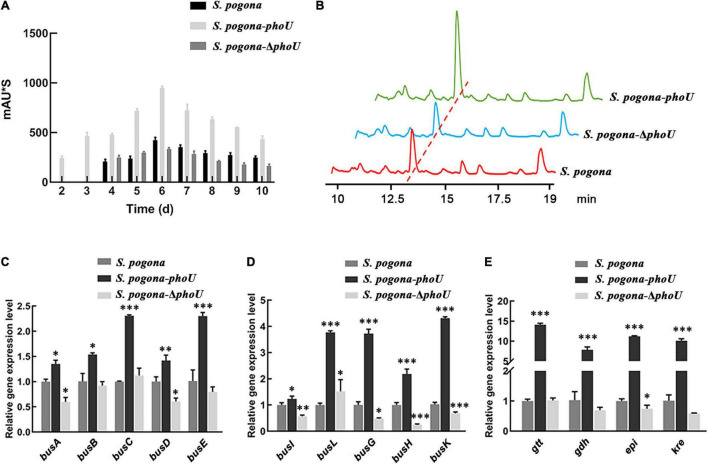
Analysis of butenyl-spinosyn yield and *bus* genes transcriptional levels. **(A)** Accumulation of butenyl-spinosyn at different time points. **(B)** Comparative analysis of butenyl-spinosyn production. The detection wavelength was set at 250 nm during analysis, and the chromatographic peak of butenyl-spinosyn appeared at 13.1 min. **(C–E)** Comparison of the transcriptional levels of *bus* genes and rhamnose biosynthesis genes (*gtt*, *gdh*, *epi*, and *kre*) between the wild-type and mutant strains. The transcription levels of the above genes were significantly increased in *Saccharopolyspora pogona*-*phoU*. In contrast, most of the genes were downregulated in *S. pogona*-Δ*phoU*. mRNA samples were isolated from the wild-type and engineered strain cells cultured for 144 h. The 16S rRNA gene was used as an internal control to quantify the relative expression of the target genes. The error bars indicate the standard deviations of three biological replicates. *, **, and *** indicate *P* < 0.05, *P* < 0.01, and *P* < 0.005, respectively, compared to *S. pogona* under the same conditions.

The butenyl-spinosyn extracted from the fermentation broth of recombinant and wild-type strains was detected by HPLC. Additionally, the MS parent ion of butenyl-spinosyn and the characteristic ion peaks of trimethylrhamnose of butenyl-spinosyn were identified by MS (Lewer et al., 2009; Supplementary Figure 4). The HPLC results showed that the butenyl-spinosyn production of S. pogona-phoU was significantly higher than that of the wild-type strain, and butenyl-spinosyn could be detected on the second day in the broth fermentation. However, S. pogona-ΔphoU did not produce detectable butenyl-spinosyn until the fourth day, which is consistent with the wild-type strain, indicating a weaker butenyl-spinosyn biosynthesis capacity than S. pogona-phoU (Figure 2A). Moreover, the HPLC results on the sixth day showed that the peak areas for S. *pogona*-*phoU* and *S. pogona*-Δ*phoU* at 13 min were 947.9 mAU*s and 331 mAU*s, respectively, which increased by 123.4% and decreased by 21.9% compared with those for *S. pogona* (Figure 2B).

To further verify the effect of PhoU on the biosynthesis of butenyl-spinosyn, the transcription levels of *bus* genes and rhamnose synthesis related genes, which are key genes for butenyl-spinosyn biosynthesis, were monitored. The transcript levels of these genes were significantly upregulated to varying degrees in *S. pogona*-*phoU*, while declined to some extent in *S. pogona*-Δ*phoU* (Figures 2C–E). The result implied that PhoU had a positive effect on the biosynthesis of butenyl-spinosyn.

### Biological Activity Assay

**FIGURE 3:**
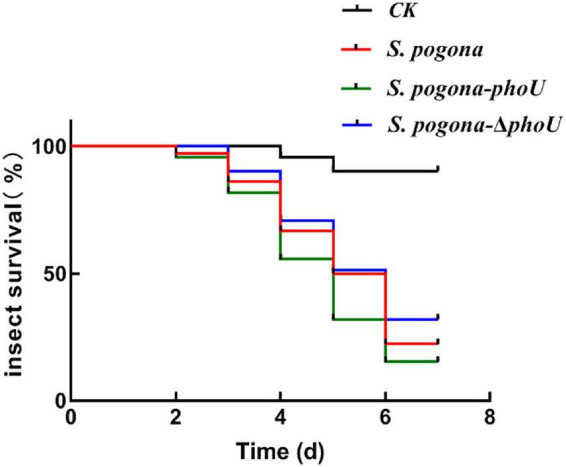
Analysis of insecticidal activity against *H. armigera*. The fermentation supernatant of *Saccharopolyspora pogona*-*phoU* showed stronger insecticidal activity than that of the wild-type and *S. pogona*-Δ*phoU*. Feed without fermentation supernatant was used as a control group.

To more intuitively verify the difference in the yield and insecticidal activity between the recombinant strains and the wild-type strain, the viability of *H. armigera* was determined after feeding with fermentation broth. From the third day, the survival rate of *H. armigera* in *S. pogona*-*phoU*-treated group was significantly lower than that in the other two groups (Figure 3), and the half lethal time (LT_50_) was also advanced by 0.55 day. In contrast, the survival rate of the *S. pogona*-Δ*phoU*-treated group was higher than that of the *S. pogona*-treated group, and the LT_50_ was delayed by 0.26 day (Table 1). The results confirmed that the deletion or overexpression of *phoU* resulted in the yield change of butenyl-spinosyn, which in turn significantly affected the insecticidal activity of the fermentation broth.

**TABLE 1:** Biological insecticidal activity of the wild-type and recombinant strains.

Strains	LT_50_ (d)	95% Confidence interval
*S. pogona*	4.831	4.592–5.104
*S. pogona*-*phoU*	4.267	4.046–4.500
*S. pogona*-Δ*phoU*	5.098	4.851–5.390

### The Effect of PhoU on the Growth and Mycelium Morphology of *Saccharopolyspora pogona*

**FIGURE 4:**
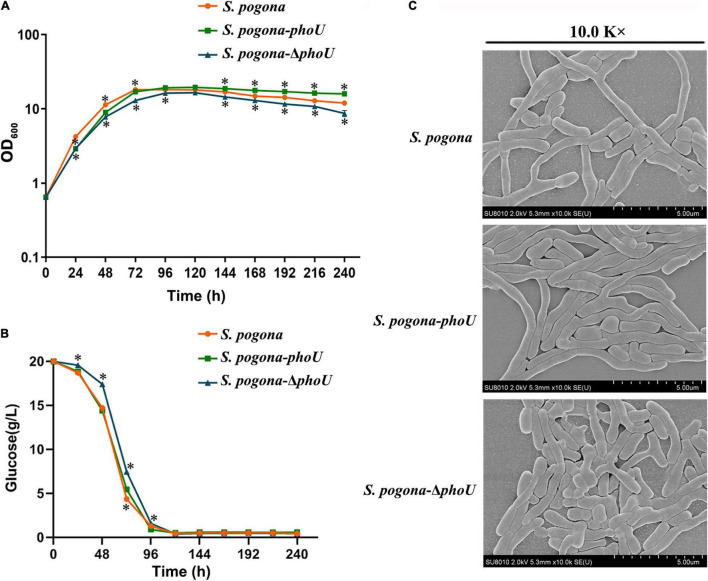
Growth and morphology differences in the wild-type, *Saccharopolyspora pogona*-Δ*phoU* and *S. pogona*-*phoU*. **(A)** Growth curves of the recombinant and wild-type strains. These growth curves are plotted using semi-log scales (linear scale for *X*-axis and log scale for *Y*-axis). The error bars indicate the standard deviations of three biological replicates. * Indicates *P* < 0.05, compared to *S. pogona* under the same conditions. **(B)** Glucose consumption of the recombinant and wild-type strains. The error bars indicate the standard deviations of three biological replicates. * indicates *P* < 0.05, compared to *S. pogona* under the same conditions. **(C)** Scanning electron microscopy observation of wild-type and engineered strains.

The growth curves of recombinant and wild-type strains were measured by UV spectrophotometry. The growth trends of the three strains were basically similar, and the stationary phase occurred at approximately 96 h. However, *S. pogona*-*phoU* showed the largest biomass, while *S. pogona*-Δ*phoU* exhibited reduced biomass and growth rate compared to wild-type and *S. pogona*-*phoU* (Figure 4A). To explore the possible causes of this phenomenon, a glucose consumption rate experiment was performed (Figure 4B). The glucose consumption trend of *S. pogona*-*phoU* was almost the same as that of the wild-type strain. Consistent with the trend of the growth curve, the glucose consumption of *S. pogona*-Δ*phoU* was significantly slower than that of the wild-type strain after about 24 h, which may be responsible for the slow growth of *S. pogona*-Δ*phoU*.

To monitor mycelium morphology and spore germination, the recombinant and wild-type strains were cultivated in CSM medium for 48 h. On one hand, the thallus from CSM medium were observed with scanning electron microscopy (SEM), on the other hand, the strains were transferred to a different solid medium to observe altered spore germination. These results revealed that *S. pogona*-*phoU* showed better transition through the growth stages than the wild-type strain, while *S. pogona*-Δ*phoU* showed shorter mycelium and fewer branches (Figure 4C). Furthermore, the spore germination experiment indicated that there was no significant difference between the recombinant and wild-type strains, suggesting that PhoU may have effects on growth and mycelial morphology, but not spore germination (Supplementary Figure 5).

### Deletion of *phoU* Resulted in an Imbalance in Intracellular Polyphosphate Levels

**FIGURE 5:**
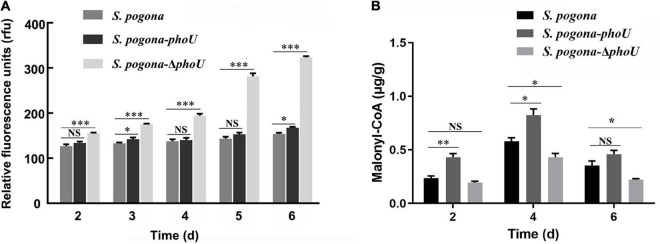
Analysis of intracellular polyP and malonyl-CoA levels in the recombinant and wild-type strains. **(A)** Intracellular polyP content in wild-type and engineered strains at different time points. **(B)** Intracellular malonyl-CoA content in wild-type and engineered strains at different time points. The error bars indicate the standard deviations of three biological replicates. *, **, and *** indicate *P* < 0.05, *P* < 0.01, and *P* < 0.005, respectively, NS, no significant difference, compared to *Saccharopolyspora pogona* under the same conditions.

To investigate possible reasons for the effect of *phoU* on the phenotype and metabolism of *S. pogona*, DAPI was used to detect intracellular inorganic polyphosphate (polyP) accumulation in different strains. PolyP is a polymer of tens to hundreds of phosphate residues linked by “high-energy” phosphoanhydride bonds as in ATP (Rao et al., 2009). It is well-known that polyP not only plays an important role in cell growth, biofilm formation and cell cycle control (Esnault et al., 2017), but also participates in the biosynthesis of secondary metabolites (Diaz et al., 2013). There was no significant difference in the level of polyP between *S. pogona*-*phoU* and *S. pogona*. However, a sharp increase in polyP levels was detected in *S. pogona*-Δ*phoU*, and this abnormal change was likely to be responsible for the strain’s growth arrest and reduced butenyl-spinosyn production (Figure 5A). The above results indicated that intracellular polyP levels were dysregulated with the inactivation of the *phoU* gene in *S. pogona*, which negatively affected cellular metabolism.

### Screening and Identification of Differentially Abundant Proteins

Previous studies have shown that PhoU, as a pivotal regulator factor, plays its role mainly by regulating the phosphate transport system PstSCAB and the two-component system PhoR/PhoP (Gardner and McCleary, 2019). To explore the regulatory mechanism of PhoU in *S. pogona*, SDS-PAGE analysis of the protein expression of the recombinant strain and the wild-type strain at different stages was performed, and the difference in total protein expression was the most significant on the 6th day (Supplementary Figure 6). Then, the distinguishing bands were extracted from an SDS-PAGE gel and identified by 1D-LC-MS/MS. The identified proteins were defined by UniProt^1^ based on functional analysis (Table 2). To further verify the differential expression of these identified proteins, the transcription levels of the relevant genes encoding these proteins were analyzed by qRT-PCR (Supplementary Figure 7).

**TABLE 2:** Differential proteins identified from SDS-PAGE gel analysis.

Serial number	MW (kDa)	Calc.pI	Score	Description
A	34.6	5.02	261.21	Malate dehydrogenase
B	35.0	5.54	141.14	(2R,3R)-2,3-butanediol dehydrogenase
C	34.6	5.11	41.65	Dihydroxyacetone kinase
D	35.2	5.05	208.00	Adenosine kinase
E	96.2	5.91	50.46	Polynucleotide kinase-phosphatase
F	26.3	8.98	194.47	TetR family transcriptional regulator

The expression levels of malate dehydrogenase (MDH) and polynucleotide kinase-phosphatase (PNKP) were downregulated in *S. pogona*-Δ*phoU*. The former catalyze the malate and oxaloacetate dependent NAD/NADH interconversion in the TCA cycle (Goward and Nicholls, 1994), and the latter is associated with DNA repair (Havali-Shahriari et al., 2017). Therefore, this result implicated inhibition of the TCA cycle and DNA damage repair, which in turn led to slower growth and reduced biomass. Acetoin is also an important precursor source for butenyl-spinosyn biosynthesis. (2R,3R)-2,3-butanediol dehydrogenase (BDH), which catalyzes the interconversion between acetoin and butanediol during pyruvate metabolism (Gonzalez et al., 2000), was upregulated in *S. pogona*-*phoU*. Moreover, dihydroxyacetone kinase (DhaK), which catalyzes the conversion of glycerol into phosphoglycerate (Gauss et al., 2018), was also upregulated, favoring the promotion of pyruvate synthesis. The increased content of acetoin and pyruvate presumably provided more malonyl-CoA precursors for the synthesis of butenyl-spinosyn, which was confirmed by the detection of intracellular malonyl-CoA content (Figure 5B). In addition, adenosine kinase (ADK), which is involved in energy metabolism, and the pivotal global regulatory factor TetR were identified in *S. pogona*-*phoU*. The upregulation of the former can provide more sufficient energy for the physiological metabolism of the strain (Estiri et al., 2018), while the latter has been confirmed to be an important positive regulator of growth and butenyl-spinosyn synthesis in *S. pogona* (He et al., 2020; Tang et al., 2021a).

**FIGURE 6:**
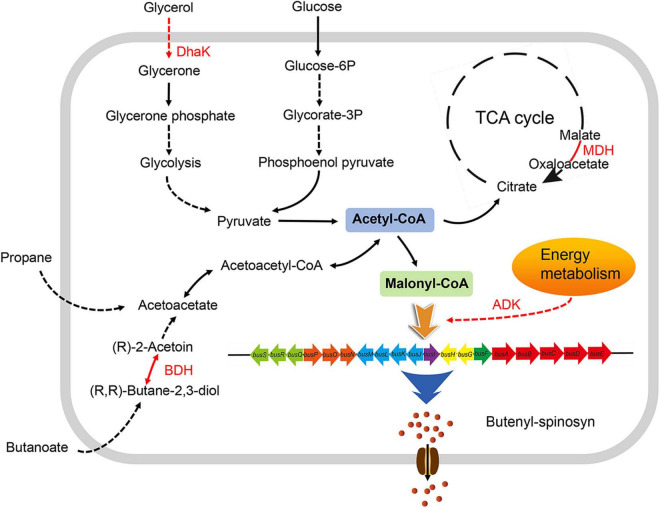
Effects of PhoU on the metabolic network in *Saccharopolyspora pogona*. The enhanced expression of DHAK and BDH in *S. pogona*-*phoU* promotes the degradation of glycerol and butanoate, which favors the synthesis of acetyl-CoA, thereby promoting the TCA cycle and the synthesis of the precursor malonyl-CoA. Enhanced expression of MDH and ADK further promotes the progression of the TCA cycle and the energy supply for butenyl-spinosyn biosynthesis. Therefore, PhoU indirectly affects glycerol and butanoate metabolism, TCA cycle, and energy metabolism *via* regulating intracellular phosphate levels, providing more malonyl-CoA precursors and energy for butenyl-spinosyn biosynthesis. Differentially expressed proteins in *S. pogona*-*phoU* are shown in red font; black arrows indicate that the process is not affected; red arrows indicate that the process is enhanced; solid arrows indicate that the process has only one-step reaction; dashed arrows indicate that multiple-step reactions are omitted in the process.

Overall, these differentially expressed proteins were likely to be important factors responsible for the significant differences in the growth and butenyl-spinosyn yield of the recombinant strains. Based on the above results and analysis, a metabolic regulatory network associated with PhoU protein was constructed by KEGG^2^ analysis (Figure 6).

## Discussion

Phosphorus is the sixth most abundant element in living organisms and is considered limiting for global primary productivity because its phosphate form is essential for life on Earth (Barreiro and Martinez-Castro, 2019). However, excess phosphate in actinomycetes can still produce obstacles to vital processes, such as impaired growth and decreased secondary metabolites production (Rang et al., 2020). Therefore, actinomycetes have evolved a series of intracellular phosphate regulatory mechanisms in the course of evolution, including the two-component system PhoR/PhoP and the phosphate regulatory protein PhoU (Ghorbel et al., 2006; Mendes et al., 2007; Fernandez-Martinez et al., 2012).

In this study, we characterized PhoU *via* constructing overexpression and knockout recombinant strains, confirming its active role in regulating the growth of *S. pogona* and the biosynthesis of butenyl-spinosyn. Growth curves and HPLC results indicated that PhoU might be a regulatory factor in cell growth and secondary metabolite synthesis in *S. pogona* (Figures 2A,B, 4A). The evidence suggested that excessive accumulation of phosphate levels produces a variety of negative effects on cells, therefore, quantification of intracellular polyP in the recombinant strains and wild-type strain was performed. The results showed that an excess level of polyP was detected in *S. pogona*-Δ*phoU*, which may be one of the factors leading to growth retardation and a decrease in butenyl-spinosyn production (Figure 5A). By distinctive protein analysis, MDH, which is the key enzyme in catalyzing the NAD/NADH conversion between malate and oxaloacetate associated with the TCA cycle, was identified. SDS-PAGE results showed that the expression of this protein was downregulated in *S. pogona*-Δ*phoU*, which inhibited the TCA cycle and slowed the metabolic rate of cells, thus leading to a slow growth rate and decreased biomass. Moreover, BDH catalyzed the interconversion between acetoin and butanediol in pyruvate metabolism and DhaK catalyzed the production of phosphoglycerides in lipid metabolism. These proteins were determined to be upregulated in *S. pogona*-*phoU*, which facilitates the supply of butenyl-spinosyn biosynthetic precursors (Figure 5B). ADK involved in energy metabolism, PNKP involved in DNA repair and fragmentation, and global regulator TetR family proteins, also showed differential expression in the recombinant strains. The expression of the ADK, DhaK and BDH proteins related to energy metabolism, lipid metabolism and pyruvate metabolism was upregulated in *S. pogona*-*phoU*, which promoted the growth and metabolism, thus increasing the biomass. Moreover, lipid metabolism and pyruvate metabolism produce a large amount of acetyl-CoA, which plays a key role in promoting butenyl-spinosyn biosynthesis as the main precursor. Downregulated expression of MDH and PNKP was detected in *S. pogona*-Δ*phoU*, which may affect DNA repair, cell division, and restricted primary metabolism, thereby impeding strain growth and butenyl-spinosyn biosynthesis (Havali-Shahriari et al., 2017).

Previous studies have shown that PhoU, as a peripheral membrane protein, works as a pivotal regulatory factor in maintaining phosphate homeostasis in cells and mainly plays a role by regulating the phosphate transport system PstSCAB and two-component system PhoR/PhoP (Gardner and McCleary, 2019). Moreover, the PhoU protein has a remarkable influence on the bacterial growth and development, intracellular polyphosphate levels (diCenzo et al., 2017; Shang et al., 2020), and secondary metabolite biosynthesis (Sola-Landa et al., 2003; Martin-Martin et al., 2018). There are two relatively accepted models to explain how PhoU is involved in signaling pathways. (I) PhoU may mediate the formation of a signaling complex between the PstSCAB transporter and PhoR (Oganesyan et al., 2005); (II) PhoU may generate a unique signaling factor recognized by the PhoR domain (Hoffer and Tommassen, 2001).

PstSCAB transporter have unique domains within their nucleotide-binding domains that enable negative feedback regulation by binding to specific proteins, a process known as transinhibition (Gerber et al., 2008; Kadaba et al., 2008; Johnson et al., 2012; Yang and Rees, 2015). When Pi is abundant, PhoU likely performs this function for the PstSCAB transporter, preventing excess Pi import with concomitant ATP hydrolysis (Rice et al., 2009). PhoU is considered as an intermediate between the Pst and Pho systems, and PhoR is inhibited when the Pst system actively transports phosphate; when phoU is mutated or deleted, PhoR is activated as an autokinase, resulting in high-level expression of Pho-regulated genes (Steed and Wanner, 1993; Baek et al., 2007). Notably, PhoP is not only a specific regulator that controls intracellular phosphate levels, but it also appears to regulate genes related to nitrogen and carbon metabolism (Rodriguez-Garcia et al., 2009). PhoP binds to the promoters of *glnR* (encoding nitrogen regulators), *glnA* and *glnII* (encoding two glutamine synthetases), and the *amtB*-*glnK*-*glnD* operon (encoding ammonium transporters and nitrogen regulators), thereby inhibiting these nitrogen metabolism genes. *phoU* deletion mutants exhibit poor growth and frequently accumulate compensatory mutations in the *phoR*, *phoB*, or *pstSCAB* genes (Steed and Wanner, 1993; Rice et al., 2009). This suggests that strain growth is compromised when intracellular ployP level is excessive. Therefore, PhoU may affect many physiological and metabolic processes by regulating the PhoR/PhoP two-component system.

In our previous study, it was found that the SenX3-RegX3 system also regulates phosphate balance in *S. pogona*. Proteomic and targeted metabolomic analysis showed that mutations in this system cause ployP imbalance, which in turn leads to changes in multiple biological processes, including glycolysis, PP pathway, TCA cycle, fatty acid metabolism, oxidative phosphorylation and amino acid metabolism (Rang et al., 2020; Rang et al., 2022). In addition, PhoU may also indirectly affect the physiological metabolism of *S. pogona* by affecting the expression of TetR family transcriptional regulators, which have also been shown to have positive effects on precursor supply processes such as central carbon metabolism and energy metabolism (He et al., 2020; Tang et al., 2021a). The above analyses may explain that PhoU indirectly affects the pathways of precursor synthesis and energy metabolism by regulating the level of intracellular ployP, thereby regulating the growth and butenyl-spinosyn biosynthesis in *S. pogona*. However, the target of PhoU direct action and the underlying regulation mechanism still need to be further studied.

In summary, the above results indicate that PhoU, as a positive regulator, regulates the growth and butenyl-spinosyn biosynthesis in *S. pogona via* regulating intracellular phosphate, which in turn affects multiple primary metabolic pathways such as TCA cycle, pyruvate metabolism and lipid metabolism (Figure 6). This study examines the function of PhoU in *S. pogona* for the first time, demonstrating its important role in maintaining phosphate balance, strain growth, and butenyl-spinosyn biosynthesis, providing a reference for improving secondary metabolites of actinomycetes.

## Data Availability Statement

The original contributions presented in the study are included in the article/Supplementary Material, further inquiries can be directed to the corresponding author.

## Ethics Statement

The animal study was reviewed and approved by the Biomedical Research Ethics Committee of Hunan Normal University.

## Author Contributions

LX, JT, and JCh were responsible for the original concept and designed the experiments. YS, ZY, and JCu analyzed the data. JT, JCh, YL, JH, ZX, XL, HH, and JR performed the experimental work. JT and JCh wrote the manuscript. All authors read and approved the final manuscript.

## Conflict of Interest

The authors declare that the research was conducted in the absence of any commercial or financial relationships that could be construed as a potential conflict of interest.

## Publisher’s Note

All claims expressed in this article are solely those of the authors and do not necessarily represent those of their affiliated organizations, or those of the publisher, the editors and the reviewers. Any product that may be evaluated in this article, or claim that may be made by its manufacturer, is not guaranteed or endorsed by the publisher.

## Footnotes

1
www.uniprot.org

2
www.kegg.jp
