# Supplementary material for: The Global Regulator PhoU Positively Controls Growth and Butenyl-Spinosyn Biosynthesis in Saccharopolyspora pogona
Source: Front Microbiol. 2022 Jun 9;13:904627. doi: 10.3389/fmicb.2022.904627 (PMC9218956; doi:10.3389/fmicb.2022.904627)
Supplement: Supplementary file 1 [file Data_Sheet_1.pdf]

## Supplementary Material

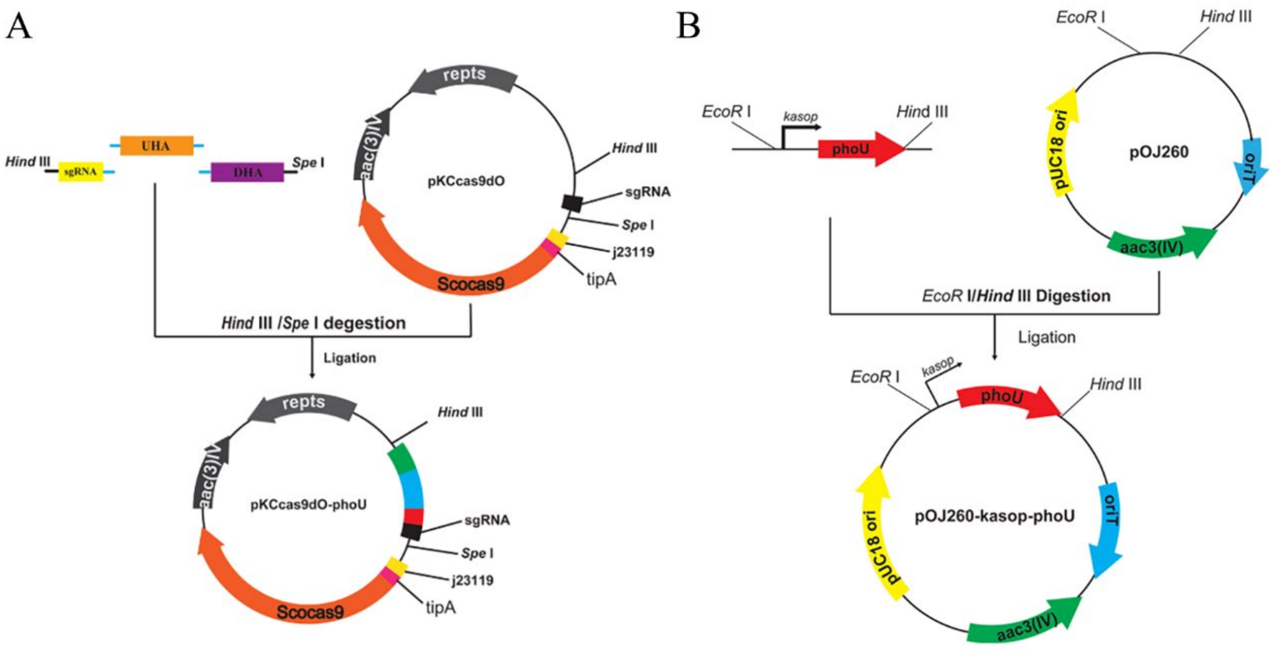


**Figure S1 | Construction process of recombinant vectors.** (A) Construction of recombinant plasmid pKCcas9dO-*phoU*. (B) Construction of recombinant plasmid pOJ260-$P_{kasO}$-*phoU*.

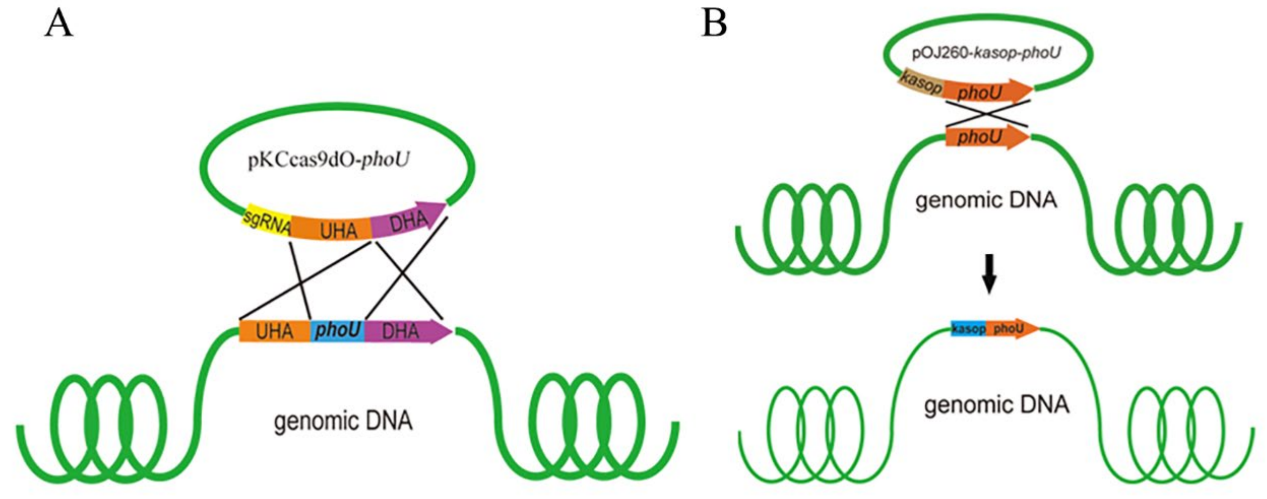


**Figure S2 | Construction process of recombinant strains.** (A) Construction of knockout strain *S. pogona*-Δ*phoU*. (B) Construction of overexpressed strain *S. pogona*-*phoU*.

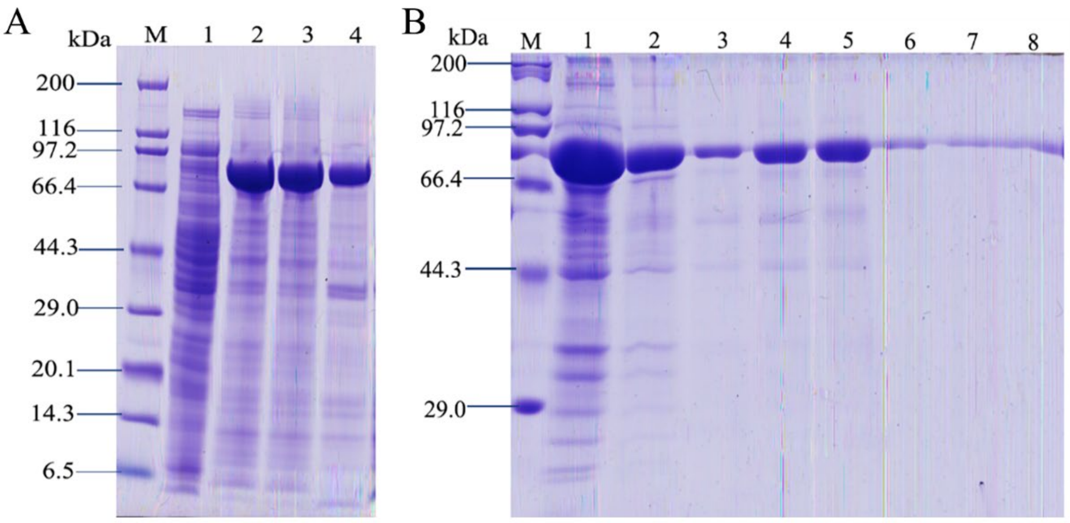


**Figure S3 | SDS-PAGE analysis of PhoU induced expression and purification.** (A) Inducible expression of PhoU protein. M: Protein Maker; 1: Not induced *E. coli* BL21-pCold-TF-*phoU*; 2-4: Induced *E. coli* BL21-pCold-TF-*phoU*. (B) Column purification of PhoU protein. M: Protein Maker; 1: Ni-Native-0; 2: Ni-Native-20; 3: Ni-Native-50; 4: Ni-Native-100; 5: Ni-Native-250; 6-8: Ni-Native-500.

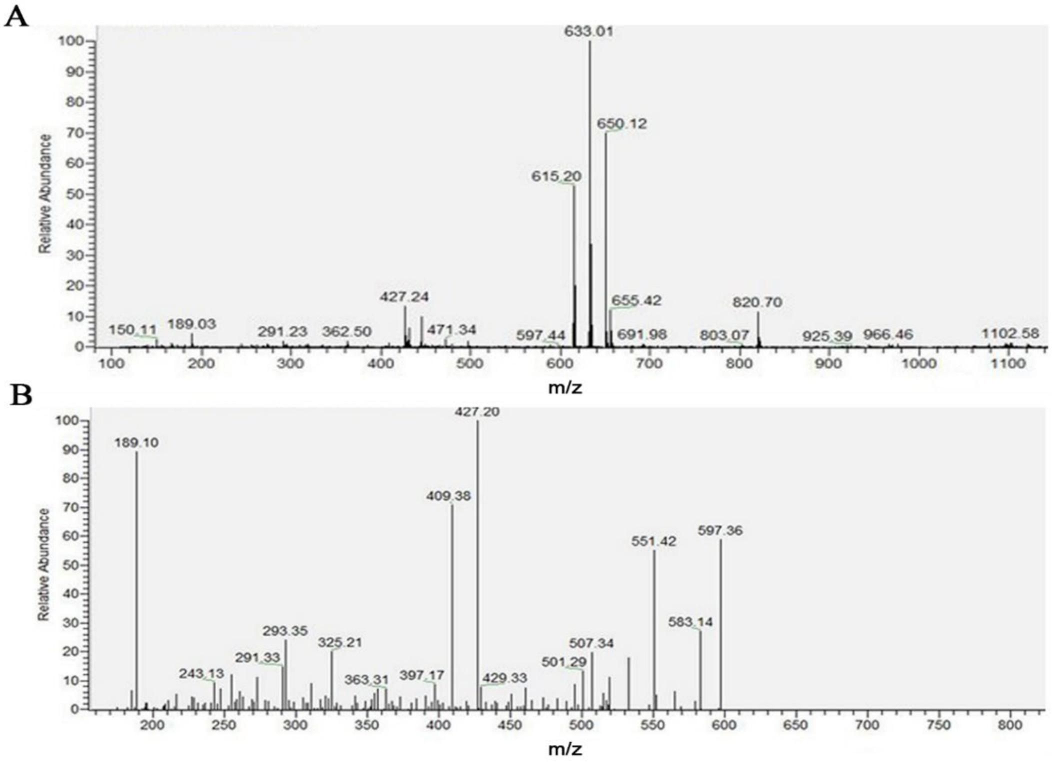


**Figure S4 | Mass spectrum identification of butenyl-spinosyn.** (A) The ESI Full ms of the sample. (B) The ESI Full ms2 of the parent ion 633.01. MS identification results indicated that full MS contained a spinosyn ion species (633.01), and the MS/MS ion contain a trimethylrhamnose fragment of 189.10 molecular mass, which was confirmed as a butenyl-spinosyn component.

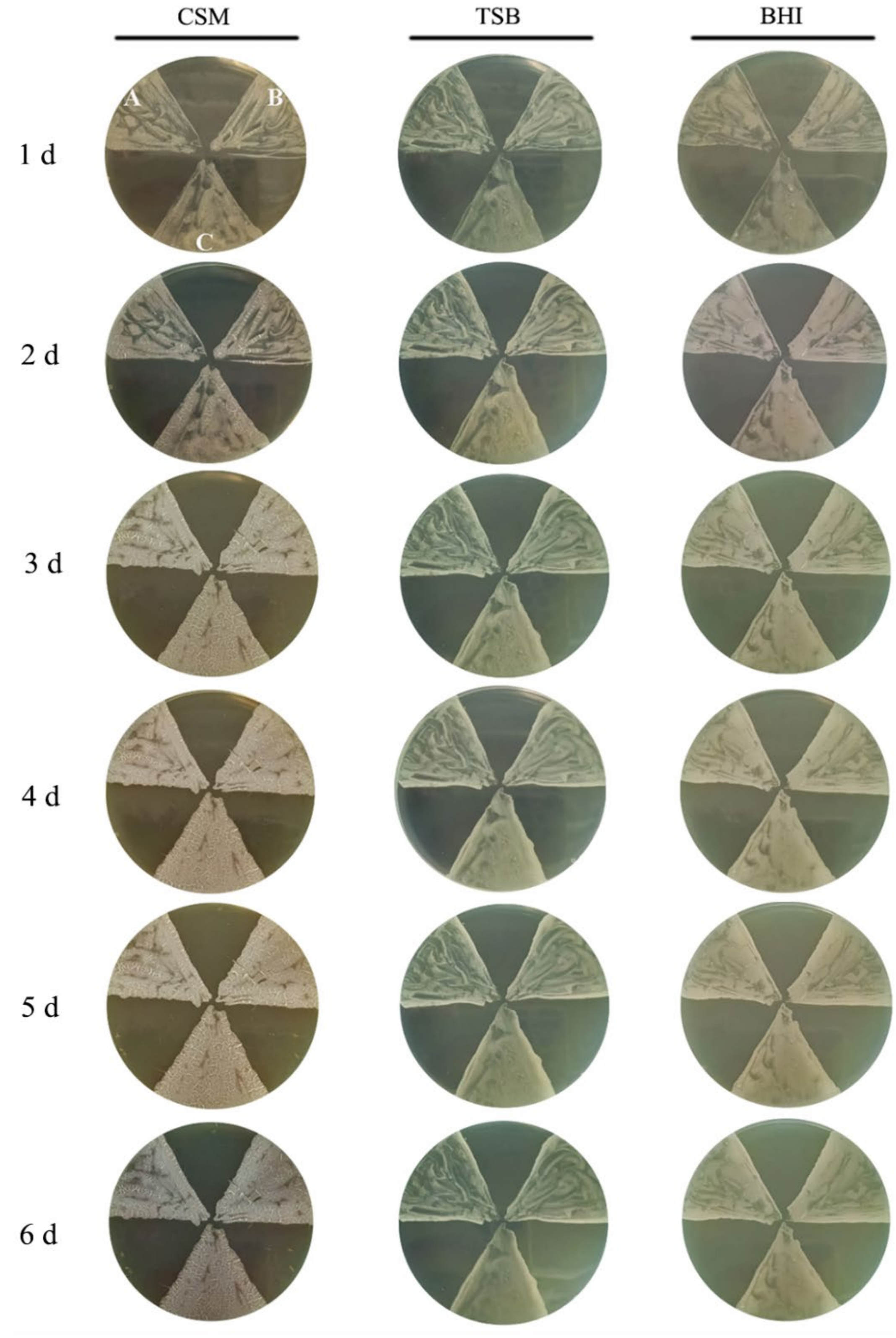


**Figure S5 | Observation of the sporulation capacity of *phoU* gene engineered strains on different solid media.** A: *S. pogona- phoU*; B: *S. pogona*-Δ*phoU*; C: *S. pogona*.

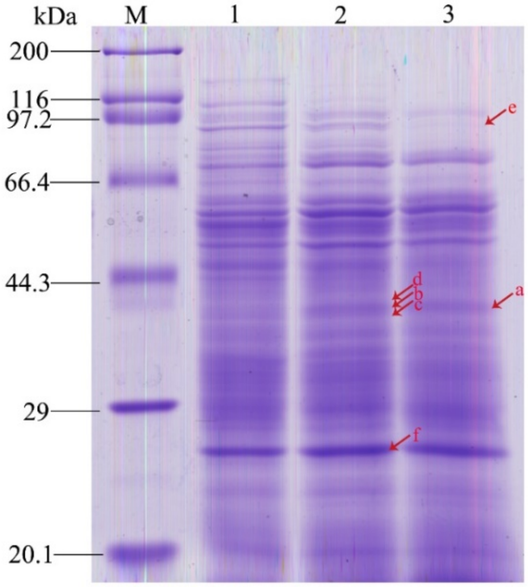


**Figure S6 | SDS-PAGE analysis of the whole protein in the wild type and engineered strains.** M: Protein marker 200.0 kDa; 1: The protein sample of *S. pogona*; 2: The protein sample of *S. pogona-phoU*; 3: The protein sample of *S. pogona*-Δ*phoU*.

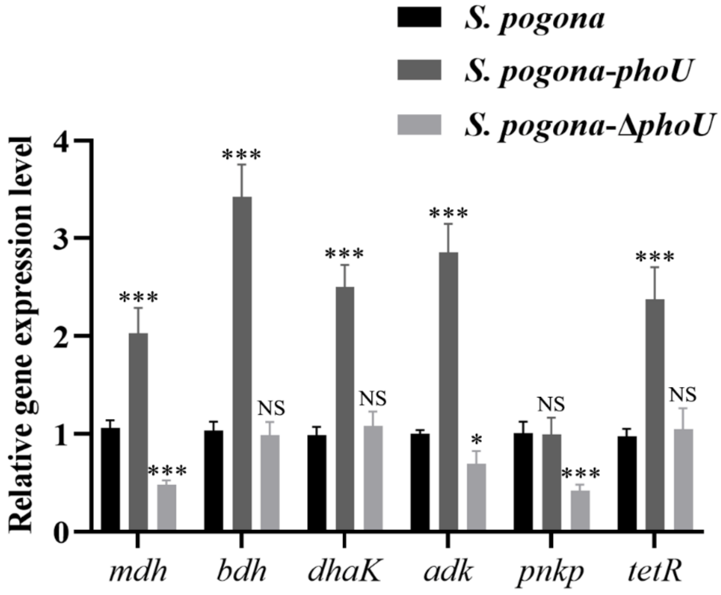


**Figure S7 | qRT-PCR analysis of genes encoding differentially expressed proteins in the engineered strains.** *mdh*, *bdh*, *dhaK*, *adk*, *pnkp*, and *tetR* represent genes encoding malate dehydrogenase, (2R,3R)-2,3-butanediol dehydrogenase, dihydroxyacetone kinase, adenosine kinase, polynucleotide kinase-phosphatase, and TetR family transcriptional regulator, respectively. The 16S rRNA gene was used as an internal control to quantify the relative expression of the target genes. The error bars indicate the standard deviations of three biological replicates. *, **and *** indicate P < 0.05, P < 0.01 and P < 0.005, respectively, NS, no significant difference, compared to *S. pogona* under the same conditions.

**TABLE S1 | Strains and plasmids**

| Strains | Relative description | Sources |
|---|---|---|
| **Strains** | | |
| *E.coli* DH5α | Host for general cloning | Lab store |
| *E. coli* S17 | Donor strains for conjugation | Lab store |
| *S. pogona* | The producer strains of butenyl-spinosyn | Lab store |
| *S. pogona-phoU* | *S. pogona* harboring pOJ260-$P_{kasO}$-*phoU* | This work |
| *S. pogona-ΔphoU* | *S. pogona* harboring pKCcas9dO-*phoU* | This work |
| *E.coli BL21*(*DE3*)-pCold-TF-*phoU* | *E. coli* BL21(DE3) containing the plasmid pCold-TF-*phoU* | This work |
| **Plasmids** | | |
| pOJ260 | *E. coli*-cloning vector, containing pUC18 replicon, oriT, AprR | Lab store |
| pKCcas9dO | *E. coli*-cloning vector, containing Scocas9, oriT, AprR | Lab store |
| pOJ260-$P_{kasO}$-*phoU* | $P_{ermE}$-*phoU* inserted into pOJ260 by *Hin*d Ⅲ and *E*coR I | This work |
| pKCcas9dO-*phoU* | Homologous arm inserted into pKCcas9dO by *Hin*d Ⅲ and *Spe* I | This work |
| pCold-TF-*phoU* | *phoU* inserted into pCold-TF by *Hin*d Ⅲ and *Xba* I | This work |

**TABLE S2 | Nucleotide sequences of primers**

| Primers | Sequence (5’→3’) |
|---|---|
| $P_{kasO}$-F | CCGGAATTCTGTTCACATTCGAACGGTCT |
| $P_{kasO}$-R | CTCACGCAAACTCCCCCAGTCCTGCACG |
| *phoU*-F | GGGAGTTTGCGTGAGGTCTACCAGGAAC |
| *phoU*-R | GCAAAGCTTCCCGTCACCTGCGAGAAC |
| *phoU*-sgRNA-F | TTGGACTAGTGGTCGGCATCTTTCCGGTCAGTTTTA GAGCTAGAAA |
| *phoU*-sgRNA-R | TACCTCAAGTCTCAAAAAAAGCACCGACTCGG |
| *phoU*-UHA-F | TTTGAGACTTGAGGTAGTCCGCCGTGTC |
| *phoU*-UHA-R | GATTCCGGTTCTCGCAGGTGACGGG |
| *phoU*-DHA-F | TGCGAGAACCGGAATCGGACAGAATGA |
| *phoU*-DHA-R | CCCAAGCTTCGCTTACCGTGTTCCTGA |
| H-*phoU*-F | CCCAAGCTTATGCGTGAGGTCTACCAG |
| H-*phoU*-R | GCATCTAGAGTCACCTGCGAGAACGGC |
| *phoU*-YZ-F | GCTGGAGTCCTTGGGGTT |
| *phoU*-YZ-R | CATCCCGTTCGATTTCGT |
| *bus*A-F | GCAACCTCCCTGGATTACGG |
| *bus*A-R | ATGAACACGCCGTATCCACC |
| *bus*B-F | CGGAGGACTTGTGGCAACTG |
| *bus*B-R | GACACGCCCTGAAGGAAACC |

| | |
|---|---|
| *bus*C-F | TGTCCGATGTGGACGTTGTG |
| *bus*C-R | GCCTGCGTATGACCGATGTT |
| *bus*D1-F | GTTGCATTTGGCGTGCCAGT |
| *bus*D1-R | ACAACCGCTCCAGCAGAACC |
| *bus*D2-F | TGAGGGTGCCGGTCTAGTGT |
| *bus*D2-R | ATTTGACAACGCCTGGGTGA |
| *bus*E-F | CGGTCTCCTGGGCAGTGATC |
| *bus*E-R | CCTCGGAAGCAACCTCCAAG |
| *bus*F-F | ACCAGGTGGACTTCTCGTGC |
| *bus*F-R | ATCCCGCTGCCTATTTCTCG |
| *bus*G-F | TCCCGCTCAACCTGTTCCTG |
| *bus*G-R | CTGCTCATCCGGCAAGCAGA |
| *bus*H-F | GACAACCTCCAGCACTGCGT |
| *bus*H-R | GGTCCGACTCGTGGTCTTGC |
| *bus*I-F | GTCCTTCCATGCCCTGTTTC |
| *bus*I-R | AGGCCGTCGATCAGTTCTTT |
| *bus*J-F | CGCGAGATGTACGCCGAAAC |
| *bus*J-R | ACAGGCCGTGCTGGAAGATG |
| *bus*K-F | ACGACGGCAGCCACATCAAC |
| *bus*K-R | ACGCCTTCCAGCAGGTTCTT |
| *bus*L-F | GCGATGCAGTCCGTGGTACA |

| | |
|---|---|
| *bus*L-R | TCAAGGTATGGGCGGTCGTG |
| *bus*M-F | CCCCACGCTCGTCTACCACA |
| *bus*M-R | CGAACCAGGAGACGACCACT |
| *bus*N-F | TGCTGCGGGAGAATTACCTG |
| *bus*N-R | TTCGGTGCGATAGCGGATGT |
| *bus*O-F | AGCAACTACACGCAGGCACA |
| *bus*O-R | CCGAGGGTCAACCAGCAGAA |
| *bus*P-F | TGCGACTGCCTGTGGACTTG |
| *bus*P-R | TGCCTGTTCCTGGGCTTCTC |
| *bus*Q-F | CCCCACGACCATCAATCCAG |
| *bus*Q-R | GATTTCGTCAGCGGCAAAGG |
| *bus*R-F | ATTCGGCCAAGTCTTCCAAC |
| *bus*R-R | CGTACTGCGTAGCGATTTCC |
| *bus*S-F | ACATCTACGACGCGATCCAC |
| *bus*S-R | TCGCATCGGACAGTTCAAGC |
| *gtt*-F | GGAGCAGCCGAAGTCCAACA |
| *gtt*-R | GTGTCCAGCCAGGCGAAACC |
| *gdh*-F | TTCATCGGCTCGCACTACGT |
| *gdh*-R | GTTCGCGGTCACAGATGTCG |
| *epi*-F | ACCGTGATGACCTACCTCTG |

| | |
|---|---|
| *epi*-R | CGGTCCTTTTCAGACAGGAT |
| *kre*-F | GGTGCTGCACTGCACCAATT |
| *kre*-R | TCGGACAGGACCGAGTAGGC |
| *mdh*-F | CATTCGACGGCACCAACGT |
| *mdh*-R | CGCCCTGCGGTTTGAAGAT |
| *bdh*-F | AGACGTGGTGTTCGAGTGCG |
| *bdh*-R | ACCGAGTCCAGTTCTGAGGGTC |
| *dhaK*-F | CGTCCTGCTGTTCACCAACTCG |
| *dhaK*-R | GGCTCATGCCCTGCATCTCC |
| *adk*-F | CGGATGGAAGGCGACGACA |
| *adk*-R | GAGCGAGGTCACCCAGATGC |
| *pnkp*-F | AGACCCACGAGATCAACAACCG |
| *pnkp*-R | TCACCCAGGAAGTAGGGCTTG |
| *tetR*-F | ATGTCCGTGCCAGTCCCGCC |
| *tetR*-R | GCACGTAGGCGTACAGCGAG |
